# Comparison of Lower Extremity Muscle Deterioration in Women with Rheumatoid Arthritis and Healthy Controls: Relationship with Disease Activity, Muscle Strength, and Walking Speed

**DOI:** 10.5152/eurasianjmed.2026.251144

**Published:** 2026-04-14

**Authors:** Merve Karapınar, Veysel Atilla Ayyıldız, Aylin Alsaç, Atalay Doğru, Mehmet Şahin

**Affiliations:** 1 Department of Physiotherapy and Rehabilitation, Süleyman Demirel University Faculty of Health Sciences, Isparta, Türkiye; 2 Kocaeli City Hospital, Kocaeli, Türkiye; 3 Manisa City Hospital, Manisa, Türkiye; 4 Department of Rheumatology, Süleyman Demirel University Faculty of Medicine, Isparta, Türkiye

**Keywords:** Disease activity, muscle quality, muscle strength, rheumatoid arthritis, walking speed

## Abstract

**Background:**

This study aimed to compare muscle quality between women with rheumatoid arthritis (RA) and age-matched healthy controls and to explore the associations with muscle strength and walking speed.

**Methods:**

Thirty women with RA and 30 healthy controls participated. Muscle thickness and echo intensity (EI) of the rectus femoris (RF), vastus medialis, tibialis anterior (TA), medial gastrocnemius (MG), and lateral gastrocnemius (LG) were assessed using B-mode ultrasonography. Disease activity was evaluated using the 28-joint Disease Activity Score. Isometric knee and ankle muscle strength were measured by handheld dynamometry and walking speed by the 20-meter Walk Test.

**Results:**

Women with RA exhibited significantly lower muscle thickness in the RF (1.08 vs. 1.51 cm; *P* = .032), TA (1.46 vs. 1.98 cm; *P* = .027), MG (1.09 vs. 1.53 cm; *P* = .040), and LG (0.86 vs. 1.13 cm; *P* = .013) compared with controls. Echo intensity values were significantly higher in the RA group across all muscles examined (all *P* < .05). Knee extensor strength (109.26 vs. 122.35 N; *P* = .004) and walking speed (1.09 vs. 1.98 m/s; *P* = .032) were also reduced in RA. Disease Activity Score correlated positively with EI (RF: rho = 0.425) and negatively with muscle thickness (RF: rho = −0.456).

**Conclusion:**

Women with RA show significant impairments in muscle quality, strength, and mobility, which worsen with increasing disease activity. Regular assessment of muscle morphology and functional performance may support early identification of functional decline and guide appropriate management strategies in this population.

**Main Points**

- Women with rheumatoid arthritis (RA) show significantly reduced muscle thickness and increased echo intensity compared to healthy controls, highlighting impaired muscle quality.
- Muscle quality correlates with muscle strength, walking speed, and disease activity, demonstrating its functional and clinical relevance.
- Altered muscle characteristics indicate early sarcopenia risk, emphasizing the importance of routine muscle assessment in RA management.

## Introduction

Rheumatoid arthritis (RA) is a chronic, systemic, inflammatory autoimmune disorder characterized by synovial inflammation and damage to cartilage and bone. It affects around 0.5%-1% of people in the general population, with a higher prevalence in women.^1^ Although RA primarily targets the joints, increasing evidence suggests that it also significantly impacts the musculoskeletal system beyond the synovium, indicating that RA should be considered a condition with broader systemic consequences.^2^

In patients with RA, increased levels of pro-inflammatory cytokines disrupt the balance between muscle damage and repair cycles, leading to changes in muscle composition and reduced muscle quality. These cytokines exacerbate inflammation, accelerate muscle deterioration, and impair regenerative capacity.^3^ Hormonal factors, particularly age-related declines in estrogen, further negatively affect muscle metabolism, strength, and quality, contributing to more pronounced muscle atrophy in women with RA.^4^ Together, these findings highlight the importance of investigating skeletal muscle alterations in this population. Understanding specific alterations in skeletal muscle, particularly in lower extremity characteristics, is essential for elucidating mechanisms underlying functional impairment and for informing targeted rehabilitation strategies aimed at improving physical performance and quality of life. Previous studies, such as that by Blum et al,^5^ have reported reductions in quadriceps muscle thickness and strength in individuals with RA compared with healthy controls; however, these assessments often focused solely on muscle size. More recent research suggests that qualitative muscle characteristics, such as echo intensity (EI) measured via ultrasound, may provide a more accurate representation of muscle composition and functional capacity than thickness alone.^6^

Despite these insights, the specific quantitative and qualitative alterations in lower extremity muscles in RA remain incompletely understood, and their relationships with disease activity, muscle strength, and walking speed have not been thoroughly explored. Ultrasound-based assessment offers a non-invasive and clinically applicable method for early detection of muscle deterioration, potentially informing more tailored rehabilitation and treatment strategies. Therefore, the aim of this study was to investigate lower extremity muscle deterioration—specifically in the rectus femoris (RF), vastus medialis (VM), medial gastrocnemius (MG), lateral gastrocnemius (LG), and tibialis anterior (TA)—in women with RA compared with age-matched healthy controls using B-mode ultrasound imaging. Additionally, the aim was to examine the relationship between alterations in muscle characteristics, walking speed, and muscle strength in patients with RA.

## Material and Methods

This study was conducted between April and September 2023 and included 30 women with RA and 30 age-matched healthy women as controls. Inclusion criteria included adults aged 20-65 years, diagnosed with RA according to established criteria, the 2010 American College of Rheumatology/European League Against Rheumatism (ACR/EULAR) classification criteria.^7^ Both healthy individuals and patients with RA were included in the study. However, individuals with muscular dystrophy, myopathy, neuropathy, spinal disorders, or diabetes; those who had active infections or arthritis; individuals with hip or knee prostheses; those diagnosed with malignancy; patients who had experienced any lower extremity injury or surgery within the past year; and individuals with communication difficulties were excluded from the study.

Written informed consent was obtained from all participants before data collection. The study was conducted in accordance with the Declaration of Helsinki and approved by the Clinical Research Ethics Committee of Süleyman Demirel University (Approval no: 8 Date: 12/01/2023). The study involved a multidisciplinary approach to assess participants with rheumatological conditions. A rheumatologist conducted medical examinations and administered patient-reported outcome measures.

The sample size was determined using data from Pan’s study.^8^ Based on the difference in skeletal muscle mass between healthy individuals and those with RA, a power analysis was performed using the G*Power 3.1 sample size calculation program with the following inputs: two-tailed independent *t*-test, effect size (ES) : 0.8 (large), power: 86%, and a 5% type 1 error rate. According to these data, a minimum of 30 participants should be included in each group.

Demographic information was collected, including age, sex, and duration of disease from onset of symptoms and medications. Disease activity in patients with RA was assessed by the 28-joint Disease Activity Score (DAS-28). The DAS-28 considers 28 tender and swollen joint counts, general health (patient assessment of disease activity using a 100 mm Visual Analog Scale with 0 = best, 100 = worst), plus levels of C-reactive protein (mg/L). The resulting DAS-28 score provides a quantitative measure of disease activity, with higher scores indicating more active disease.^9^

Longitudinal ultrasound images were obtained using musculoskeletal B-mode ultrasound (Phillips EPIQ 7 180 Plus) with a 10 MHz linear transducer for measurements of muscle characteristics in key lower limb muscles, including the RF, VM, MG, LG, and TA. A gain of 48 dB and a dynamic range of 84 dB were applied for the measurements performed on all muscles. This assessment focused on determining muscle thickness and echogenicity to provide comprehensive insights into muscular characteristics. All ultrasonographic assessments were performed by a rheumatologist with 10 years of experience and expertise in musculoskeletal ultrasound.

During the ultrasonographic evaluation, participants were positioned in a supine position for the RF and VM muscles; participants were typically positioned in a supine position with hip and knee fully extended and the ankle in the neutral position. RF and VM muscle images were recorded at each marking (50% of the thigh length).^10^ The MG and LG were evaluated with participants in a prone position, while the TA muscle was assessed with participants lying supine.^11^ While the TA muscle was assessed at 30% proximal between the lateral malleolus of the fibula and the lateral condyle of the tibia, the MG and LG muscles were evaluated at 30% proximal between the lateral malleolus of the fibula and the lateral condyle of the tibia.

For the assessment of the muscle thickness, the distance between deep and superficial aponeuroses was considered, and it was calculated through the mean value of 3 parallel lines drawn at right angles between the superficial and deep aponeuroses along each ultrasonography image.^7^

Assessments of muscle EI were conducted using grayscale histogram analysis using the ImageJ program version 1.52a (National Institutes of Health, Maryland, USA). Higher mean grayscale values (0-255 arbitrary units) were indicative of lower muscle quality.^12^^,^^13^ A rectangular marquee tool was utilized to select a region of interest within the superior and inferior fascicle borders, along with the lateral field of view, and mean grayscale units were then averaged from 3 separate measurements.^14^^,^^15^

Isometric knee and ankle muscle strength was assessed using the “make test,” whereby the examiner held the Hand-Held Dynamometer stationary while the participants actively exerted a maximal force.^16^ For the isometric knee extensor muscle strength test, each participant was seated on the edge of a bed with hips and knees flexed at 90 degrees. To assess isometric muscle strength in the quadriceps femoris, the handheld dynamometer was placed perpendicular to the tibia, approximately 1-2 cm above the lateral malleolus.^17^ For the isometric ankle flexor and extensor muscle strength test, participants were seated with hips flexed and knees extended, feet positioned over the edge of an examination table. The Hand-Held Dynamometer (HHD) was placed against the metatarsal heads on the plantar surface of the foot to measure plantar flexion, and on the dorsal aspect of the foot proximal to the metatarsal heads to measure dorsiflexion.^18^ Prior to starting the test, each participant received verbal instructions about the testing procedure. They were instructed to exert maximal force against the dynamometer. Measurements were repeated 3 times with a 10-second rest period between each repetition. Each muscle group contraction lasted for 5 seconds. All testing procedures were conducted by a single examiner (M.K.).

The timed 20-meter walk test was utilized to assess the average walking speed of participants. This self-paced assessment method is recognized for its validity and reliability in gauging both average walking speed and functional performance in adults with arthritis.^19^ A 20-meter pathway was clearly marked with distinct starting and ending points. Participants were instructed to cover the course at their normal walking speed and to maintain their speed until they reached the finish point. Each participant was subjected to 3 trials of the test and mean values were obtained from these trials.

### Data Analysis

Descriptive characteristics were summarized with mean/SD for continuous variables and frequency counts for categorical variables. Normality was confirmed by visual analysis of histograms and the Shapiro–Wilk test. Since the data were not normally distributed, median and min-max values are presented. Mann–Whitney *U*-test was used to compare muscle characteristics, muscle strength, and walking speed in both groups. In the analysis using the Mann–Whitney *U*-test, the ES was calculated using the formula *r* = *z*/√n. The ES is interpreted as small if it is <0.3, moderate if it is between 0.3 and 0.5, and large if it is >0.5.^20^ Relationships between muscle characteristics in the more-involved limb and variables such as patient characteristics (age, body mass index [BMI], disease duration), DAS-28, and walking speed were measured by Spearman correlation coefficients. If the Spearman correlation coefficient (rho) value is <0.2, it indicates a very weak correlation. A value between 0.2 and 0.4 is interpreted as a weak correlation, a value between 0.4 and 0.6 as a moderate correlation, and a value between 0.6 and 0.8 as a high correlation, and >0.8 as a very high correlation. All data analyses were performed using SPSS Statistics 28 (IBM, Armonk, NY, USA). The significance level was set at *P* ≤ .05 for all analyses.^21^

## Results

Thirty women with RA and 30 age-matched healthy women were included in the study. Demographic and clinical features data are described in Table 1. The muscle thickness of 5 different muscles was measured. The median thickness of the RF was 1.08 cm in women with RA and 1.51 cm in healthy women, showing a statistically significant difference (*P* = .012) with an ES of 0.5. For the VM, women with RA had a median thickness of 2.52 cm versus 2.88 cm in healthy women, with no significant difference (*P* > .05). The TA showed a median thickness of 1.46 cm in women with RA, significantly lower than the 1.98 cm in healthy women (*P* = .027, ES = 0.6). The MG in women with RA had a median thickness of 1.09 cm, significantly less than the 1.53 cm in healthy women (*P* = .04, ES = 0.7). For the LG, women with RA had a median thickness of 0.86 cm which was significantly lower than the 1.13 cm observed in healthy women (*P* = .013, ES = 0.5) (Table 2).

Additionally, muscle EI, indicating muscle quality, was measured. The RF in women with RA had a median EI of 85.45 au, higher than the 71.65 au in healthy women (*P* = .040, ES = 0.7). For the VM, women with RA showed a median EI of 87.08 au compared to 79.44 au in healthy women, which was statistically significant (*P* = .032, ES = 0.4). The TA had a median EI of 85.33 au in women with RA, higher than the 72.49 au in healthy women (*P* = .017, ES = 0.5). For the MG, women with RA had a median EI of 85.54 au compared to 79.80 au in healthy women, which was statistically significant (*P* = .002, ES = 0.3). The LG also showed a statistically significant difference in EI between women with RA (87.46 au) and healthy women (82.04 au), with *P* = .042 and ES = 0.3 (Table 2).

The isometric knee extensor strength was 109.26 N in women with RA compared to 122.35 N in healthy women (*P* = .004, ES = 0.8). For isometric ankle extensors, women with RA had a strength of 40.23 N versus 54.12 N in healthy women (*P* = .012, ES = 0.9). Isometric ankle flexor strength was 34.26 N in women with RA, significantly lower than 46.12 N in healthy women (*P* = .023, ES = 0.8). Lastly, the 20-meter walking test showed that women with RA took a median time of 1.09 m/s compared to 1.98 m/s for healthy women, with a significant difference (*P* = .032, ES = 0.6) (Table 2).

Age showed significant negative moderate correlations with the thickness of several muscles, including RF (rho = −0.455), VM (rho = −0.348), MG (rho = −0.651), and LG (rho = −0.506) (*P* < .05). However, there was no significant relationship between age and the thickness of the TA muscle (*P* > .05). There was also no significant correlation between BMI and muscle thickness (*P* > .05). However, BMI exhibited a positive weak to moderate relationship with the EI of muscles: RF (rho = 0.235), VM (rho = 0.432), and MG (rho = 0.643) (*P* < .05) (Table 3).

Disease duration was significantly positively correlated with the EI of muscles, but not with their thickness. Specifically, for RF, there was a significant positive moderate correlation with EI (rho = 0.455, *P* < .05) but no correlation with thickness. Similarly, MG showed a weak positive correlation with EI (rho = 0.211, *P* < .05), but not with thickness. LG also had a significant weak positive correlation with EI (rho = 0.347, *P* < .05) and no correlation with thickness. No significant correlations were found between disease duration and either the thickness or EI of VM and TA muscles (*P* > .05) (Table 3).

Disease activity showed a significant positive moderate correlation with muscle EI and a negative weak correlation with thickness in several muscles: RF (thickness rho = −0.456, EI rho = 0.425), MG (thickness rho = −0.224, EI rho = 0.363), and LG (thickness rho = −0.332, EI rho = 0.356) (*P* < .05). Isometric knee extensor muscle strength was highly positively correlated with RF muscle thickness (rho = 0.663) and negatively correlated with RF EI (rho = −0.762). Similarly, it correlated moderately positively with VM muscle thickness (rho = 0.511) and negatively with VM EI (rho = −0.674). Isometric ankle extensor muscle strength was moderately to highly negatively correlated with the EI of MG (rho = −0.673) and LG (rho = −0.539). Isometric ankle flexor muscle strength was highly positively correlated with TA muscle thickness (rho = 0.680) and moderately negatively correlated with TA EI (rho = −0.584) (Table 3).

The 20-meter walking speed was moderately to highly positively correlated with the thickness of several muscles: RF (rho = 0.532), VM (rho = 0.223), TA (rho = 0.785), MG (rho = 0.795), and LG (rho = 0.653). Additionally, walking speed was weak to highly negatively correlated with the EI of these muscles: RF (rho = −0.612), VM (rho = −0.643), TA (rho = −0.321), MG (rho = −0.332), and LG (rho = −0.401) (Table 3).

## Discussion

The findings of this study provide valuable insights into lower extremity muscle quality and functional impairments in women with RA compared to healthy individuals. These results demonstrated that women with RA exhibited reduced muscle thickness and increased muscle EI relative to healthy controls. In addition, women with RA showed decreased muscle strength and slower walking speed, which were significantly associated with disease severity and duration. These findings highlight the importance of assessing muscle quality, including both muscle thickness and EI, in clinical evaluations of RA patients. Improving muscle quality may provide a crucial strategy for preventing or mitigating declines in physical performance among women with RA.

Muscle thickness significantly decreased with age in several muscles (RF, VM, MG, and LG) in women with RA, reflecting age-related atrophy exacerbated by chronic inflammation and reduced physical activity. Santos et al^22^ reported an RF thickness of approximately 1.23 cm in patients with RA, consistent with the current study’s findings. Baker et al^23^ also reported reduced skeletal muscle mass density in RA compared to healthy controls. Interestingly, TA thickness was not significantly associated with age, suggesting it may be less affected by aging in RA. Recent studies emphasize the importance of assessing muscle quality to monitor sarcopenia in RA.^24^ While thresholds for muscle thickness, such as 3.6 cm for RF and vastus intermedius in women with RA, have been proposed, combining muscle EI and thickness provides a stronger ultrasonographic predictor of sarcopenia.^25^^,^^26^ Sarcopenia is estimated to affect ~31% of rheumatic patients, yet it remains underdiagnosed. Although sarcopenia was not directly assessed, these results demonstrate poorer muscle quality in RA compared to healthy controls.

Although this study did not measure systemic inflammatory markers or mitochondrial function, previous studies suggest that pro-inflammatory cytokines, particularly Tumor Necrosis Factor (TNF)-α and Interleukin (IL)-6, may contribute to muscle alterations by activating catabolic pathways, inhibiting myogenic differentiation, impairing mitochondrial function, and promoting intramuscular fat infiltration.^27^ These mechanisms could help explain the observed relationship between disease activity and impaired muscle morphology reported in the literature.

While BMI did not correlate significantly with muscle thickness, it positively correlated with EI for several muscles (RF, VM, and MG). Pan et al^8^ also demonstrated that patients with both early and established RA had lower BMI and decreased skeletal muscle mass compared to matched controls. This suggests that higher BMI in women with RA is associated with poorer muscle quality, characterized by increased intramuscular fat and fibrosis. These results highlight the importance of managing body composition to maintain muscle health in RA. It is important to understand the impact of disease activity on muscle tissue in patients with RA. Higher disease activity is associated with decreased muscle thickness and increased muscle EI in muscles such as RF, MG, and LG in this study. These associations highlight potential negative effects on muscle function and health. Farrow et al^2^ also reported that muscle damage happened in patients with RA regardless of disease activity. Therefore, there is a need to develop strategies to preserve and enhance muscle health in the management of RA.

In this study, higher disease activity was associated with reduced muscle thickness and increased EI in muscles such as the RF, MG, and LG, reflecting potential deterioration in muscle architecture and quality. Although these findings support the hypothesis that ongoing inflammation negatively affects muscle morphology, previous studies have reported inconsistent results.^2^These discrepancies may be due to differences in disease duration, sample characteristics, and assessment methods, such as ultrasonography versus Dual-energy X-ray absorptiometry (DXA) or bioimpedance noting that ultrasonographic measurement of muscle EI is operator-dependent and no inter-observer reliability assessment was performed, which should be considered in future studies. Nevertheless, several studies indicate that higher disease activity correlates with reduced muscle mass, increased echogenicity, and lower strength, and cohort studies have reported that RA patients with higher DAS-28 or C-reactive protein levels exhibit greater sarcopenia prevalence and reduced muscle strength.^28^^,^^29^ Taken together, these findings suggest that disease activity can influence muscle quality, although the relationship may be modulated by multiple patient- and method-related factors. Farrow et al^2^ also reported that muscle damage can occur in patients with RA regardless of disease activity, reinforcing the need to develop strategies to preserve and enhance muscle health in RA management.

Isometric muscle strength correlated significantly with muscle thickness and EI. Knee extensor strength was positively associated with RF and VM thickness and negatively with their EI, while ankle extensor and flexor strength showed similar patterns with MG, LG, and TA. These findings suggest that poorer muscle quality, including increased intramuscular fat, may impair neuromuscular activation and reduce strength, consistent with prior reports linking higher EI to lower Surface Electromyography (sEMG) amplitude.^30^^,^^31^Walking speed in the 20-meter test was positively associated with muscle thickness and negatively with EI, indicating that higher-quality muscles support better functional performance. Lusa et al^32^ reported that age, psychosocial factors (e.g., depression, fatigue, pain), articular features, and body composition together explained nearly 61% of the variation in walking speed. Notably, articular features accounted for only a small portion of this variation, suggesting that muscle quality—which was not analyzed in their study—may be a key determinant of physical function in RA.

This study has several important limitations. The exclusive inclusion of female participants may limit the generalizability of the findings to broader populations, including males, and the relatively small sample size reduces statistical power, highlighting the need for larger cohorts in future research. The cross-sectional design prevents causal inferences, allowing only the identification of associations. Additionally, information regarding patient medications (e.g., Disease-Modifying Anti-Rheumatic Drugs or biologic therapies), which can influence both inflammation and muscle mass, was not reported. Finally, the measurement of muscle EI is operator-dependent, and no inter-observer reliability assessment was conducted; incorporating such assessments in future studies would strengthen methodological rigor. Despite these limitations, the study provides valuable insights into muscle quality in RA.

This study provides valuable insights into lower extremity muscle quality and functional impairments in women with RA. These findings indicate that compared to healthy individuals, women with RA exhibit lower muscle thickness and increased muscle EI. Furthermore, decreased muscle strength and walking speed were observed, which were significantly associated with the severity and duration of the disease. Aging was also shown to exacerbate declines in muscle quality, correlating with reduced lower extremity muscle strength and walking speed. These findings underscore the importance of assessing muscle quality in RA patients and highlight a critical perspective for enhancing physical performance.

## Figures and Tables

**Table 1.** Demographic Characteristics in Participants

	**Rheumatoid Arthritis, Median (Min-Max)**	**Healthy Controls, Median (Min-Max)**	***P***
Age (years)	49.50 (41-53)	50 (38-56)	.178
Height (cm)	162 (157-165)	159 (158-168)	.293
Weight (kg)	70.5 (59-74)	65 (56-70)	.613
BMI (kg/m^2^)	29.3 (20.1-30.2)	28.14 (22.12-29.46)	.342
Comorbidities			–
Diabetes (type 1 or 2) (Y : N)	12 : 18	14 : 16	
Cardiovascular diseases (Y : N)	8 : 22	7 : 23	
Thyroid disease (Y : N)	2 : 28	1 : 29	
Disease duration (months)	102 (42-150)	NA	–
Positive rheumatoid factor, n (%)	12 (50)	NA	–
Extra-articular involvement, n (%)	4 (13.3)	NA	–
Glucocorticoids dose (mg/day)	3 (0-4.5)	NA	
DAS-28	3.06 (2.81-4.67)	NA	–

BMI, body mass index; cm, centimeter; DAS-28, Disease Activity Score; kg, kilograms; m, meter; mg, milligram; N, not present; Y, present; NA; Not Applicable.

**Table 2.** Lower Extremity Muscle Thickness, Echo Intensity, Muscle Strength and Walking Speed in Rheumatoid Arthritis and Healthy Women

	**Rheumatoid Arthritis Group, Median (Min-Max)**	**Healthy Group, Median (Min-Max) **	***P***	**ES**
Muscle thickness (cm)				
Rektus femoris	1.08 (0.93-1.29)	1.51 (1.02-2.44)	.012*	**0.5**
Vastus medialis	2.52 (2.12-3.08)	2.88 (2.22-3.11)	.674	0.6
Tibialis anterior	1.46 (1.36-2.28)	1.98 (1.73-2.98)	.027*	**0.6**
Gastrocnemius medialis	1.26 (1.10-1.56)	1.53 (0.95-2.22)	.040*	**0.7**
Gastrocnemius lateralis	0.86 (0.93-1.19)	1.13 (0.76-2.00)	.013*	**0.5**
Muscle echo intensity (au)				
Rektus femoris	85.45 (75.01-93.47)	71.65 (65.22-74.30)	.040*	**0.7**
Vastus medialis	87.08 (82.26-92.10)	79.44 (72.87-95.65)	.032*	**0.4**
Tibialis anterior	85.33 (79.49-90.04)	72.49 (68.67-86.37)	.017*	**0.5**
Medial gastrocnemius	85.54 (76.46-91.56)	79.80 (75.60-85.15)	.002*	**0.3**
Lateral gastrocnemius	89.46(83.42-93.69)	82.04 (72.65-92.07)	.042*	**0.3**
Muscle strength (NN)				
Isometric knee extensors	109.26(86.12-112.34)	122.35(98.43-132.34)	.004*	**0.8**
Isometric ankle extensors	40.23 (42.45-72.43)	54.12(42.31-76.99)	.012*	**0.9**
Isometric ankle flexors	34.26 (30.11-53.45)	46.12 (34.32-60.39)	.023*	**0.8**
20-meter walking test (m/sec)	1.09 (0.7-2.27)	1.98 (1.2-3.60)	.032*	**0.6**

au, arbitrary unit; cm, centimeters; ES, effect size; m, meter; Nm, Newton–Meters; Sec, seconds.

*Bold values indicate statistically significant differences (*P* < 0.05).

**Table 3.** Correlations Between Lower Extremity Muscle Characteristics and Age, Body Mass Index, Disease Duration and Activity, Muscle Strength, and Walking Speed in Patients with Rheumatoid Arthritis

	**Rectus Femoris**		**Vastus Medialis**		**Tibialis Anterior**		**Medial Gastrocnemius**		**Lateral Gastrocnemius**	
	**Thickness**	**Echo Intensity**	**Thickness**	**Echo Intensity**	**Thickness**	**Echo Intensity**	**Thickness**	**Echo Intensity**	**Thickness**	**Echo Intensity**
**Age (years) **	−0.455**	0.563**	−0.348**	0.592*	−0.437	0.483*	−0.651**	0.784**	−0.506*	0.597**
**BMI**	0.653	0.235*	0.567	0.432*	0.355	0.453	0.436	0.643*	0.212	0.653
**Disease duration**	0.421	0.455*	0.456	0.124	0.764	0.322	0.764	0.211*	0.135	0.347*
**DAS-28**	−0.456*	0.425*	-0.673	0.567	−0.431	0.678	−0.224*	0.363*	−0.332*	0.356*
**Isometric muscle strength**										
Isometric knee extensors	0.663*	−0.762**	0.511*	−0.674**	0.367	−0.568	0.645	−0.542	0.432	−0.365
Isometric ankle extensors	0.445	0.373	0.542	0.486	0.737	0.439	0.562	−0.673*	0.432	−0.539*
Isometric ankle flexors	0.724	0.229	0.679	0.672	0.680*	−0.584*	0.432	0.356	0.385	0.830
**20-meter walking speed**	0.532*	−0.612**	0.223*	−0.643*	0.785*	−0.321*	0.795*	−0.332*	0.653*	−0.401*

BMI, body mass index; DAS-28, Disease Activity Score.

*P < .05.

**P < .001.
